# Sexuality, intimacy, and body image among adolescents and young adults with cancer: a qualitative, explorative study

**DOI:** 10.1007/s00520-024-08405-6

**Published:** 2024-03-09

**Authors:** Line Bentsen, Maria Aagesen, Pernille Bidstrup, Maiken Hjerming, Helle Pappot

**Affiliations:** 1 Department of Oncology, Copenhagen University Hospital, Rigshospitalet, Blegdamsvej 9, 2100 Copenhagen, Denmark; 2 Occupational Science, User Perspectives and Community-Based Interventions, Department of Public Health, University of Southern Denmark, Odense, Denmark; 3 Psychological Aspects of Cancer, Danish Cancer Society Research Center, Strandboulevarden 49 2100, Copenhagen, Denmark; 4 Department of Hematology, Copenhagen University Hospital, Rigshospitalet, Blegdamsvej 9, 2100 Copenhagen, Denmark

**Keywords:** Adolescents and young adults, AYAs, Cancer, Sexual health, Intimacy, Body image

## Abstract

**Aim:**

The aim of this study was threefold: (1) to explore Danish adolescents and young adults’ (AYAs) thoughts concerning sexual health particularly focusing on sexuality, intimacy, and body image throughout a cancer trajectory, (2) to investigate how AYAs experience healthcare professionals address of- and respond to sexual health issues, and (3) to identify AYAs’ suggestions on how to support conversation about sexual health.

**Methods:**

A qualitative, single-center study was conducted, including AYAs (18–29 years) diagnosed with cancer recruited at the University Hospital of Copenhagen, Rigshospitalet. Individual semi-structured interviews were conducted from January–February 2023, recorded, transcribed verbatim, and analyzed using thematic analysis.

**Results:**

Twelve participants were interviewed, aged 20–29; five were diagnosed with hematological- and seven with oncological cancer. Our analyses yielded three themes: (1) sexuality and body image as part of the identity, (2) excluding relatives in conversations about sexual health, and (3) uncertainty how to discuss sexual health with healthcare professionals. Finally, the AYAs’ suggestions to support conversations about sexual health were organized into six thematic categories.

**Conclusion:**

In this study, participants experienced altered sexual subsequent impacts on body image and self-esteem during their cancer trajectory. While some adapted to these changes, discussing them with healthcare providers was difficult, especially in the presence of relatives, as the AYAs wanted to shield them from additional concerns. To enhance support, AYAs suggest regular discussions on sexual health and the use of a dialog tool by healthcare professionals.

**Supplementary Information:**

The online version contains supplementary material available at 10.1007/s00520-024-08405-6.

## Introduction

Globally, there are approximately 1.2 million new cancer cases yearly among adolescents and young adults, typically defined as individuals aged 15 to 39 years (AYAs) [1]. AYAs experience cancer during a critical period of life characterized by personal and social development, including exploration of sexual self-awareness, desires, intimacy, sexual and romantic relationships, and sexual identities [2–5]. Due to cancer and cancer treatment, AYAs face complex age-specific physical and psychological challenges, which may directly or indirectly impact physical, emotional, mental, and social aspects of their sexual health [6–11].

During treatment, chemotherapy may reduce hormones linked to libido. For males, radiation and surgery in the genital area may result in changes to blood vessels or nerves, leading to issues like erectile dysfunction. For women, in addition to radiation and surgery, anti-hormone treatment may cause vaginal dryness and atrophy [6]. Indirectly, for both male and females, treatment side effects such as fatigue may leave little energy for sex, and weight loss or gain as well as scars may impact body image and attractiveness negatively affecting sexual health [6, 12, 13]. These side effects may persist after cancer treatment as late-effects [6]. Communication regarding potential late-effects can be challenging within a clinical setting, influenced by a range of factor [14–18]. Previous research uncovering the consequences of cancer and its treatment has primarily relied on quantitative studies with a cross-sectional design [19–21]. Consequently, there is a need for more in-depth research to explore the perspectives of AYAs with cancer concerning their sexual health.

Although qualitative studies on sexual health in AYAs with cancer have been conducted in the United States and United Kingdom [19–22], more knowledge is needed from countries with different sexual culture and norms e.g., Denmark. This is due to the fact, that the social, economic, and political context plays a crucial role in shaping our understanding and expression of sexual health, leading to cross-cultural variations [11, 23].

The aim of this study was threefold: (1) to explore Danish AYAs thoughts concerning sexual health with a particular focus on sexuality, intimacy, and body image throughout their cancer trajectory, (2) to investigate how AYAs experience that their healthcare professionals address and respond to sexual health issues, and (3) to identify AYAs’ suggestions on how to support conversation about sexual health.

## Methods

### Design and setting

This qualitative study employs a phenomenological approach to explore the thoughts of AYAs with cancer regarding sexuality, intimacy, and body image throughout their cancer trajectory. The study was carried out at the University Hospital of Copenhagen, Rigshospitalet, from January to February 2023. To ensure methodological rigor and comprehensive reporting, the study adhered to the COnsolidated criteria for REporting Qualitative research Checklist in its execution and subsequent reporting of findings [24] (see Supplementary file 1).

### Participants and recruitment

Inclusion criteria were: AYAs diagnosed with cancer within the age range 18–29 years within the last five years. Purposive sampling was employed to ensure diversity in terms of gender, age, cancer diagnosis, and relationship status. Recruitment was conducted through a closed Facebook group created specifically for AYAs connected to “Kræftværket,” a youth support center for AYAs with cancer at the University Hospital of Copenhagen, Rigshospitalet.

### Data collection

Twelve individual face-to-face semi-structured interviews were conducted by the first authors, comprising a female medical doctor Ph.D. student (L.B.) and a female physiotherapist Ph.D. student (M.A.) with prior experience conducting qualitative interviews. The interviewers did not engage in the participants’ cancer trajectory. The utilization of individual semi-structured interviews provided a platform for participants to openly express their thoughts and share personal experiences regarding sexuality, intimacy, and body image throughout their cancer trajectory [25]. Furthermore, the approach facilitated in-depth exploration enrichening the data [25]. The interviews followed a semi-structured interview guide outlined in Table 1. The guide was designed in alignment with the study’s aim and with inspiration from the EORTC SHQ-22 [26] and the Danish, national survey (Project SEXUS) [27]. All interviews took place in a quiet office at the University Hospital of Copenhagen, Rigshospitalet. The location of interviews was, since the participants were recruited from Kræftværket, situated at the Department of Oncology at the same hospital. We wanted to secure a safe environment at the same department where the participants received treatment or were in follow-up. If the participants wanted to be interviewed at another location, we offered the possibility.

**Table 1:** The semi-structured interview guide

Main topic	Examples of follow-up questions
Thoughts about sexuality	• Could you describe how satisfied you were with your sex life before cancer diagnosis? • Could you describe how satisfied you have been with your sex life after a cancer diagnosis? • Could you please elaborate if you have experienced concerns about sexuality, intimacy, and body perception? • For example, have you been worried that sexual activity could cause pain? • For example, have you been afraid of not being able to find a sexual partner? • Could you describe if you experienced sexual dysfunction after a cancer diagnosis? • Only males: Could you please elaborate if you have felt confident in achieving and maintaining an erection during sex? • Only males: Could you please elaborate if you have felt less masculine due to your cancer and the treatment? • Only females: Could you please elaborate if you have experienced vaginal dryness during sexual activity? • Only females: Could you please elaborate if you have felt less feminine due to your cancer and the treatment?
Thoughts about intimacy	• Could you please describe your perception of intimacy? • Could you please elaborate on your thoughts about intimacy when you were diagnosed with cancer? • Has your need for intimacy changed since you were diagnosed with cancer? • How do you experience intimacy after being diagnosed with cancer?
Thoughts about body image	• Could you please describe how satisfied you are overall with your appearance? • Could you please elaborate on how you perceived/thought about your body before you were diagnosed with cancer? • Could you please elaborate on if you have or still experience changes in your body and its appearance since being diagnosed with cancer and why you think you experience this change?
Involvement of relatives regarding sexual health	• Could you please elaborate if you have felt or feel the need for having some family members or partner present during the conversation about sexual health? • Did you find it difficult to discuss these topics if family members or partners were present?
Communication about sexual health with healthcare professionals	• Could you please elaborate on if you have had discussions about sexual health/sexual dysfunction/intimacy with healthcare professionals during your cancer trajectory, and if yes, how this was initiated and what you talked about? • Can you please suggest how you would like to communicate with healthcare professionals about sexual health? How can healthcare professionals improve in this area?

The interviews were audio-recorded and had an average duration of 28 min, ranging from 14 to 42 min.

### Data analysis

Audio recordings were transcribed verbatim, with each participant assigned an I.D. number to protect their identity. Thematic analysis was used to analyze data, drawing from Malterud’s systematic text condensation [28] and Braun and Clark’s reflexive thematic analysis [29, 30].

Two authors (L.B. and M.A.) independently familiarized themselves with the data by reading all transcripts consecutively line by line as the interviews were conducted. After ten interviews, the authors recognized the same themes occurring and after 12 interviews no further new themes appeared, in accordance with the concept of data saturation [31].

Subsequently, they coded each transcript inductively, identifying themes related to AYAs’ thoughts on sexual health, their experiences with healthcare professionals addressing these issues, and suggestions to support the conversation about sexual health. The two authors then collaborated iteratively, categorizing similar themes, and resolving disagreements until a consensus was reached. Subsequently, the remaining three authors (M.H., P.B., H.P.) each independently reviewed three transcripts line by line. All authors then discussed to reach a consensus on the themes and suggestions. Afterward, L.B. and M.A. re-read the transcripts to extract relevant quotations and data related to these themes and suggestions. Lastly, a final list of main themes and sub-themes was outlined.

### Ethics

In accordance with the Declaration of Helsinki, all participants were provided with written and verbal information regarding the aim and design of the study prior to participation. Informed written consent was obtained from all participants, and they were informed that they could withdraw their consent at any time without treatment consequences. The local Data Protection Agency of University Hospital of Copenhagen, Rigshospitalet approved the study (Journal number: P-2022–902). Due to Danish law ethical permission was not obliged for this type of study.

## Results

 Participant had a mean age of 25 years and half were in a relationship (Table 2).

**Table 2:** Demographic and cancer related clinical data of the participants (*n* = 12)

	*n*
Gender	
Female	7
Male	5
Mean age, y (range)	25,1 (22–29)
Sexual orientation	
Heterosexual	10
Bisexual	2
Relationship (yes)	6
Highest educational degree^a^	
University/university college	9
Senior high school	3
Cancer type	
Oncological cancer^b^	8
Hematological cancer^c^	4
Time since diagnosis	
Under 1 years	2
1–2 years	8
Over 2 years	2
Treatment received^d^	
Chemotherapy	11
Radiation	5
Surgery	8
Treatment status	
In treatment	4
Post treatment	8

^a^The classification is based on International Standard Classification of Education (ISCED 97)

^b^Including adrenal carcinoma, breast-, gynecological-, brain-, and testicular cancer

^c^Including leukemia and lymphoma

^d^Multiple answers were possible

### Themes regarding AYAs’ thoughts on sexual health and how they experience healthcare professionals address and respond to these issues

**Fig. 1:**
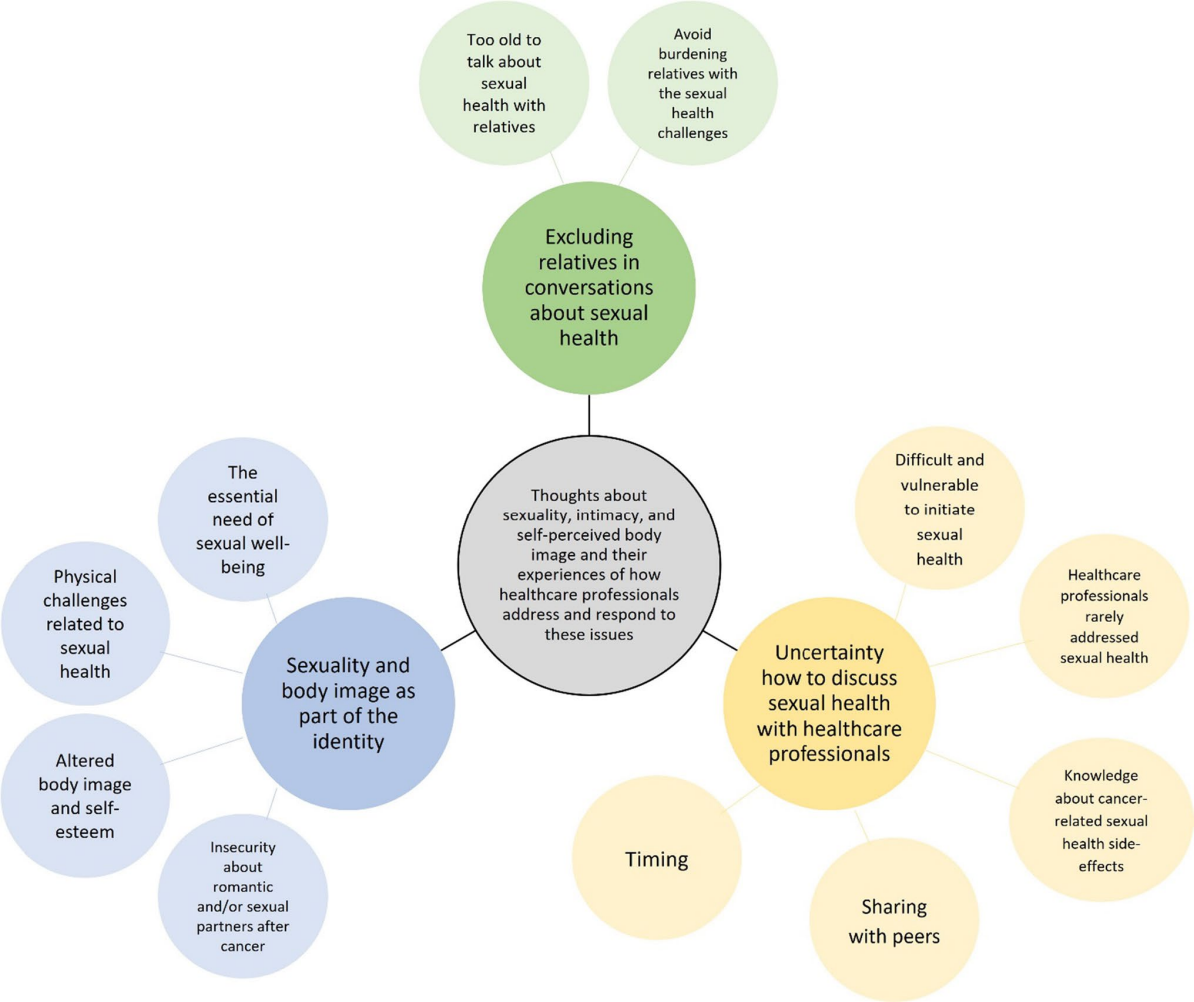
Themes and subthemes identified from the twelve qualitative interviews

Figure 1 depicts an overview of the three main themes and related subthemes derived from the data.

### Theme 1: “Sexuality and body image as part of the identity”

#### The essential need of sexual well-being

A functioning, active sex life was deemed important by all participants. Most participants explained having an active sex life and expressed contentment with it before their cancer diagnosis. Furthermore, several expressed that they would like to continue having an active sex life after their cancer diagnosis (Table 3, Quote 1). In contrast, a single participant experienced that an active sex life had become secondary after the cancer diagnosis.

**Table 3:** Themes, subthemes, and quotes

Themes	Subthemes	Quotes
Sexuality and body image as part of the identity	*The essential need of sexual well-being*	"A good sex life is a part of life. It's quite an important part of the hierarchy of needs" (Interview 7).
	*Physical challenges related to sexual health*	"Now I can't have sex. Now I can't be with anyone. Subsequently (ed. cancer treatment), I've had some side effects that affect both my fertility and my ability to have sex... I can't ejaculate anymore. And that has, of course, meant a lot to me" (Interview 6).
		"I think it has become a priority because I can't have the same amount of sex that I used to. It simply hurts too much. So maybe I appreciate intimacy more now than I did in the past" (Interview 12).
	*Altered body image and self-esteem*	"I don't look like myself and I'm not satisfied with seeing myself in the mirror. And it's not just about my weight and hair. I have four scars from my cancer. Every time I must shower or undress, I'm reminded of the cancer. I avoid having sex because I simply don't feel attractive... I have a hard time accepting myself and seeing myself and thinking that I am pretty enough for someone else" (Interview 11).
		"I find it very difficult to be an attractive man. I believe that many men have negative sexual experiences (ed. after cancer). Embodying a stereotypical, masculine role requires the ability to use one's body. That has changed because it wasn't like that before... I have become less masculine" (Interview 10).
	*Insecurity about romantic and/or sexual partners after cancer*	"It's because of my breast surgery. Because my boyfriend can't look at it... I feel less feminine because I can't wear any sexy lingerie either. It affects me a lot... Even though he expresses that he still loves me and shows affection, it doesn't change my thoughts" (Interview 5).
		"It's something that has occupied my mind. What's the deal with potential partners, potential relationships in the future? Suddenly, you must talk to them about it. If I have a night out and bring a girl home. Okay, but I have one testicle. When should I mention it? I think it would be a big deal that I can't ejaculate. It's just constantly on my mind" (Interview 6).
		"I no longer know my body, and the intimacy and security that come with knowing oneself, I no longer have that" (Interview 1).
Excluding relatives in conversations about sexual health	*Too old to talk about sexual health with relatives*	"The surgeon stops by just before my operation to make sure I know that I might experience lack of ejaculation after the operation.' I didn't know that, but I don't inquire further because my mother is there. After the surgery I had the side effect. It was extremely difficult. The situation couldn't be worse... My oncologist wants to follow up on it. But the problem is, again, my mother is there. And I don't think it's any of her business... So, I lie. I don't want my mother to know. It was terrible" (Interview 6).
	*Avoid burdening relatives with the sexual health challenges*	"When talking to healthcare professionals, you can open up about some things that you might not want to discuss with your loved ones because there are things that could upset them... And I don't believe that if I ever must get counseling from a doctor, my mother should sit next to me, because it would affect her just as much, and then it would also affect me twice as much if I had to hear something about my sex life" (Interview 11).
Uncertainty how to discuss sexual health with healthcare professionals	*Difficult and vulnerable to initiate sexual health*	"I wish that healthcare professionals would initiate the conversation because I find it really intrusive to tell a doctor that I have issues with no libido and partnership... I wish that it was a nurse who reached out to me and asked how things are going. If you don't want to answer, you can always say thanks but no thanks. But I think that as a young person, it's hard to bring up that topic" (Interview 11).
		"Such private matters can also be challenging talk about if you're constantly meeting new doctors. But if you have the same doctor, hopefully, a more confidential relationship develops, which allows you to start over with a new doctor every time" (Interview 4).
		"In consultations with my doctor, he was sort of like, 'how are you doing'? And then when I asked him about sexuality and sterility, he shut the conversation down and the topic was pushed away" (Interview 4).
	*Healthcare professionals rarely addressed sexual health*	"My mucous membranes are so dry so sometimes I get fissures. This makes it even harder to have sex. So, if something could alleviate that... my doctor for the past two years has never asked about it, and I hold back from asking. Even though at all other times, I think it's great to have the same doctor" (Interview 12).
		"At my latest consultation we had a conversation about sex. I can't quite remember exactly what we talked about, but I do remember that it was a good conversation" (Interview 4).
	*Knowledge about cancer related sexual health side effects*	"I'm very proactive when I seek answers to my questions. But you can also search online, however, it's difficult." (Interview 7)
		"In the beginning of my cancer treatment I was told not to have sex or at least not without a condom because of chemo. It could somehow be transmitted if you didn't use a condom. And I've been a bit unsure about this... I haven't talked much with anyone about sex, only with the nurse who mentioned the transmission of cancer" (Interview 2).
		"It calmed me down immensely to come to the youth facility centre and learn that there were others who felt the same way as me. I believe that if you asked everyone there if they had been nervous about sexuality after treatment, I seriously think that over half of them would raise their hands" (Interview 11).
	*Sharing with peers*	"My experience is that it's so intimate to explain about sexual health to you (ed. the interviewer) or other people. You can't relate to it unless you've experienced it. It's only us who can recognize it. So, it has created an intimacy between us with cancer" (Interview 1).
		"I've been lucky to randomly meet someone at the youth facility center, who actually knew about a testicular cancer group on Facebook. Otherwise, I wouldn't have talked to a guy, who lifted my spirits" (Interview 4).
		"It's challenging to be one of the few who sometimes have different needs. It can be tough. The youth facility centre is good for young people when there are many who have the same problem. But sometimes you can feel lonely when you don't share the same experience" (Interview 12).
	*Timing*	"It would be really great if the doctors had a small questionnaire or at least three themes about sexual health. And then there were some keywords under each theme that popped up every three months" (Interview 3).

#### Physical challenges related to sexual health

All participants described experiencing physical challenges related to sexual health under and/or after their cancer treatment. For the male participants, these challenges typically involved erection dysfunction and lack of ejaculation. The physical difficulties lead to concerns about engaging in sexually active (Table 3, Quote 2). Several participants shared that their erection dysfunction was remedied with medication. They did not experience any embarrassment associated with the need for medication. However, one concern was that obtaining prescriptions from their general practitioner was difficult and this often led to the inconvenience of contacting the oncology department for medical prescriptions.

Female participants described that the physical challenges were especially related to vaginal dryness due to antihormonal treatment, pain, and fatigue. These side effects decreased their libido, influencing their sexuality. However, several female participants expressed difficulties improving their decreased libido due to a lack of knowledge on reducing pain or fatigue. Furthermore, some female participants who were involved in romantic relationships shared that their level of intimacy increased due to factors such as a lack of energy or experiencing too much pain during sex. As a result, these participants prioritized intimacy to a greater extent than before cancer, making them feel closer to their partner (Table 3, Quote 3).

#### Altered body image and self-esteem

The physical challenges experienced by the participants in this study gave rise to mental concerns among the AYAs, particularly related to their perceived attractiveness during and after their cancer trajectory. Several participants expressed those bodily changes resulting from cancer, such as hair loss, weight gain, scars, gynecological surgery, or mastectomy, repressed their sexual desire and self-esteem. These bodily changes were a constant reminder of their cancer trajectory (Table 3, Quote 4). Furthermore, numerous male and female participants felt respectively less masculine or feminine due to their cancer treatment (Table 3, Quote 5).

#### Insecurity about romantic and/or sexual partners after cancer

The fear of not being a sufficient partner emerged among most of the participants. Particularly, if they would not perform sexually as they did before their cancer trajectory and compared themselves to peers without cancer (Table 3, Quote 6). In addition, some participants thought a lot about whether their current partner would consider leaving them due to their sexual challenges. Single participants also expressed concerns about how future partners would perceive their cancer diagnosis and feared potential rejection (Table 3, Quote 7).

For several participants, fertility concerns were closely linked to sexuality, and they found it challenging separating sexuality and fertility concerns when discussing future romantic and sexual relationships. However, some participants described a process where they tried to accept their sexual challenges over time instead of holding on to their frustration, including fertility concerns and their altered body image (Table 3, Quote 8).

### Theme 2: “Excluding relatives in conversations about sexual health”

#### Too old to talk about sexual health with relatives

Two participants with cancer in the reproductive organs did not mind discussing sexual health with relatives, primarily parents, because it was obvious that their sexuality was hampered due to the cancer. However, most participants expressed a strong preference for not discussing it with their parents. They perceived it as a private matter not concerning them. Two participants felt forced to discuss sexual challenges with healthcare professionals with their parents present at consultation, which was extremely uncomfortable. One of the participants thought he had to lie to the doctor to avoid involving his mother in this matter (Table 3, Quote 9).

#### Avoid burdening relatives with sexual health challenges

In addition to the private nature of AYAs’ sexual health, it was found that some participants argued that they did not want to burden their parents or partners unnecessarily. Conversations about sexual health were often closely tied to fertility, and several participants did not want to add further worries to their relatives regarding future risk of infertility (Table 3, Quote 10).

### Theme 3: “Uncertainty how to discuss sexual health with healthcare professionals”

#### Difficult and vulnerable to initiate sexual health

All participants openly shared information regarding their sexual health. However, during the interviews, talking about the sexual health challenges caused many participants to become upset because it was a sensitive topic. The majority explained that they wanted to discuss sexual challenges during their cancer trajectory with healthcare professionals. Nevertheless, they expressed uncertainty about how to do this because of lack of available information and resources (Table 3, Quote 11).

They also explained that they had experienced various barriers regarding addressing sexual health. It was difficult for the participants to initiate the conversation about sexual challenges, especially because they often met a new healthcare professional at every consultation (Table 3, Quote 12). If the participants initiated a conversation about sexual challenges, some experienced that the conversation was “shut down” by the healthcare professionals (Table 3, Quote 13).

#### Healthcare professionals rarely addressed sexual health

Several participants described that healthcare professionals rarely initiated conversation about their sexual health. One participant had a strong therapeutic relationship with her doctor for over the course of a year. Yet, she recalled that the doctor only once initiated a conversation about possible sexual challenges related to her cancer diagnosis. The participant expressed that she wanted to address the subject, but was afraid of potential awkwardness, as sexual challenges had never been addressed before. She expressed that it seemed too late to broach the topic as they knew each other too well at that point (Table 3, Quote 14). In contrast, for one participant, his doctor initiated the conversation about sexual health, and the participant appreciated this (Table 3, Quote 15).

#### Knowledge about cancer-related sexual health side effects

Most participants emphasized a huge need to be informed about the cancer-related side effects that could impact their sexual health. However, they felt difficulties accessing this knowledge as the healthcare professionals did not inform them systematically, neither verbally nor by handing out written materials tailored to them. Consequently, several participants searched for information online, which led them to knowledge of varying quality. The available information was often too general or too specific to relate to their situation (Table 3, Quote 16). More troublesome, one participant experienced receiving misinformation when talking to a healthcare professional about the risk of sexual transfer of cancer, making the participant believe that the cancer could be transferred through sexual activity to the partner (Table 3, Quote 17).

In contrast, the participants experienced that the youth support coordinators were knowledgeable of cancer-related side effects and were consistently available to offer advice. For instance, they provided valuable information about assistive devices to improve sexual challenges. Also, the participants mentioned that meeting the youth support coordinators and the youth facility center, Kræftværket, was a turning point on conversing about sexual health (Table 3, Quote 18).

#### Sharing with peers

Most participants emphasized that they appreciated discussing sexual health with other AYAs with cancer. This helped normalizing and decreased the feeling that they were the only AYA with cancer-related sexual challenges. Only when they could mirror themselves as equals it became much easier to discuss their sexual concerns openly (Table 3, Quote 19). Still, many participants explained that their opportunities to meet equals and discuss sexual health were mostly by coincidence (Table 3, Quote 20). One participant commented that discussing sexual health is easier if you experience the same challenges. However, if your challenges distinguish from the others, it can still be difficult to discuss, and one can feel alone and “left out” (Table 3, Quote 21).

#### Timing

Timing was deemed important to the participants when addressing sexual health with healthcare professionals. Some experienced that they had briefly been asked once about sexual health. The timing was not right for the conversation, and the topic was never brought up again. Several participants emphasized a need for a systematic approach to addressing sexual health (Table 3, Quote 22). Finally, most participants would prefer that the healthcare professionals repeatedly ask the participants about sexual health and let the participants decide whether this was the right timing.

#### AYAs’ suggestions on how to support conversation about sexual health

Table 4 shows the six suggestions that derived from the ideas AYAs put forth to support conversations about sexual health. These suggestions address the main barriers described in the three themes.

**Table 4:** AYAs’ suggestions on how to support conversation about sexual health

Suggestions	
Initiate sexual health conversations	• Inform about the possibilities to discuss sexual health with healthcare professionals several times during a cancer trajectory • Healthcare professionals should continuously ask AYA’s if they have concerns regarding sexual health • Integrate reminders at certain time points regarding addressing sexual health into the journal system
Use a dialog tool systematically	• Use predefined topics addressing various aspects of sexual health
Offer sexual health consultations	• Secure the possibilities of additional consultations with the purpose on only sexual health issues • Should be without relatives
Ensure a safe environment	• Sexual health issues should be addressed by healthcare professionals who knows the AYA
Provide referrals	• Make appropriate referrals to other healthcare professionals or support e.g., sexologists, psychologists, or support groups
Access to information	Content • Information regarding the potential impact of cancer and its treatment on sexual health • Awareness of energy conservation for maintaining energy and vitality for sexual activities • Knowledge about assisted devices e.g., use of lubricants Form • Educational videos e.g., through social media • Q&A, theme sessions • Written information disturbed as pamphlets and via E-boks^a^

*AYA*, adolescents and young adults. ^a^ is a digital mailbox in Denmark used by Danish citizens to receive correspondence from public authorities, banks, insurance companies, and other entities

## Discussion

This study yielded three themes regarding Danish AYAs’ thoughts concerning sexual health; (1) sexuality and body image as part of the identity, (2) excluding relatives in conversations about sexual health, and (3) uncertainty how to discuss sexual health with healthcare professionals. Finally, six suggestions on how to support conversation about sexual health were proposed by the AYAs.

Our results stressed that sexuality and body image were linked to the AYA’s identity and confirmed existing knowledge that AYAs experience challenges, changes, and shifts related to their sexual health during the cancer trajectory [10, 13, 18, 21, 32]. Others also argue the risk of decreased sexual satisfaction due to these challenges [9, 33]. In contrast to this, some of our participants enabled with time to accept the challenges and adapt to their new sexual health circumstances. This phenomenon aligns with the concept of response shift in quality-of-life research, where patients over time learn to cope with e.g., new symptoms. Despite persistence of the symptoms, patients often report increase in quality of life over time [34].

Males and females experience challenges related to sexual health—for males, this was especially erectile dysfunction and problems with ejaculation. For females, vaginal dryness and atrophy appeared often. The findings physical changes resemble previous findings [33, 35]. Both genders experienced change in body image and they felt less feminine or masculine due to their cancer and its treatments. This engendered insecurity when engaging in romantic and/or sexual relationships. Graugaard et al. [22] reported in a cross-sectional study that 23.6% of AYAs with cancer (*N* = 151) experienced no discernible alterations in desires, flirtation, or romantic partnership after their cancer diagnosis while 40.6% of the participants perceived a positive change in their relationship after their cancer diagnosis [22]. We found that a positive change due to cancer was mainly related to intimacy.

Overall, the participants preferred refraining from involving relatives in conversations concerning sexual health. In line with our findings, others have found that AYAs and healthcare professionals consider the parents’ presence as a barrier to initiate conversations about sexual health [16, 36], because AYAs perceive their sexual health as a private matter. For many participants in our study, sexual health issues were closely tied to concerns about fertility. Therefore, we observed another barrier in discussing sexual health issues with relatives, as AYAs sought to shield their relatives from additional worries regarding the potential risk of infertility. Similar to others, AYAs in our study lacked “split visit” consultations [37] and suggested dedicated consultations focusing solely on sexual health to ensure that discussions about sexual health are held in privacy, without the presence of relatives.

Most participants experienced that sexual health issues were rarely addressed by healthcare professionals, despite the AYAs’ need to receive information about how cancer impacts sexual health and desire to discuss how to manage their sexual health issues. This finding is aligned with results from a cross-sectional study (*N* = 56) by Albers et al. [36] who found that only 41% of AYAs did receive information about sexual health from a healthcare professional, and only 21% found the information satisfactory. Also, a nationwide population-based study (*N* = 1010) by Bergström et al. [38] reported that men to a higher extent than women reported having received information about the potential cancer-related impact on their sex life (68% vs. 54%, *p* < 0.001). The AYAs with cancer participating in our study provide suggestions for what and how to provide information about sexual health (see Table 4).

In our study, some participants experienced that if they initiated a conversation about sexual health, the topic was quickly shut down. Both Perez et al. [17] and Moules et al. [39] describe sexual health as a taboo issue for healthcare professionals in relation to AYAs with cancer. Graugaard et al. [22] also portray the “2-way taboo” where conversations related to sexual health seem difficult for both the AYAs with cancer and healthcare professionals. Finally, Frederick et al. [15] found in a cross-sectional study that conversations about sexual health initiated by healthcare professionals often focused on medical issues such as contraception.

A qualitative study (*N* = 14) by Albers et al. [40], interviewing healthcare professionals, stated that they were responsible for facilitating AYA’s sexual health needs. Still, they experienced barriers including finding the right timing, clear roles of responsibility to address the topic, and lack of education [40]. Our study also found that the timing to discuss sexual health was an important factor. In line with others, it was, however, difficult for the AYAs to state when the optimal time was [16]. Nonetheless, repeatedly addressing the topic throughout the cancer trajectory was suggested. Furthermore, AYAs suggest using predefined topics when discussing sexual health to ensure all areas are covered. Greimel et al. [41] have validated a generic questionnaire, “Sexual Health 22,” (SH22), addressing potential sexual issues affecting quality of life. To our knowledge no AYA specific questionnaire on sexual health exist and it no previous studies have examined if the SH22 adequately address AYAs sexual health.

Discussing sexual health with peers with cancer was emphasized as an advantage, making the AYAs feel more “normal.” However, other research has revealed that peer-to-peer also has some disadvantages, as AYAs, through peer-to-peer support, may be confronted with peers’ adverse events, increasing anxiety and fears [42–44]. In addition, some AYAs refrain from peer-to-peer support as they do not want their cancer disease to be a part of their narrative [42, 44]. Such disadvantages were not apparent in the present Danish study. This may be because all participants were recruited from a youth support center and social organization for AYAs with cancer, involving a high degree of peer-to-peer support or can be grounded in cultural differences.

### Further implications

Comprehending AYAs’ perspectives on sexuality, intimacy, and body image, along with their insights on supporting sexual health discussions, may enhance healthcare professionals’ ability to address AYA’s sexual health needs. This also support the need of developing nationwide guidelines at to help health care professionals to include this topic in the consultation during the cancer trajectory. Furthermore, a validated questionnaire or patient concern inventory identifying age-specific sexual health is needed to improve patient-health care provider dialog and research. As for now, the European Organization for Research and Treatment of Cancer (EORTC) has developed a quality-of-life questionnaire to assess sexual health in cancer patients. However, the questionnaire is not age specific [41].

### Strength and limitations

A strength in this study is its diverse sample, which encompassed a wide range of AYAs with varying genders, ages, cancer diagnoses, and relationship statuses. However, a limitation is that participants were exclusively recruited from a single peer-support center. We cannot exclude that the participants were more resourceful compared to other AYAs with cancer. These possible limitations can potentially lead to selection bias and reduce the external validity.

## Conclusion

Participants in this study experienced changes in sexual health during their cancer trajectory, with lasting effects on body image and self-esteem. However, some participants accepted the changes over time. Initiating conversations with healthcare professionals proved challenging due to various factors, including the presence of relatives, as the AYAs wanted to shield them from additional concerns. To improve support regarding sexual health, AYAs suggest healthcare professionals consistently invite AYAs to discuss this and consider using a systematic dialog tool.

### Supplementary Information

Below is the link to the electronic supplementary material.

Supplementary file1 (PDF 440 KB)

## Data Availability

The data generated and analyzed in this study is not publicly available due to the Danish data protection rules but could be available from the corresponding author on reasonable request.

## References

[1] Bray F, Ferlay J, Soerjomataram I, Siegel RL, Torre LA, Jemal A (2018). Global cancer statistics 2018: GLOBOCAN estimates of incidence and mortality worldwide for 36 cancers in 185 countries. CA Cancer J Clin.

[2] Arnett JJ (2000). Emerging adulthood. A theory of development from the late teens through the twenties. Am Psychol.

[3] Warner EL, Kent EE, Trevino KM, Parsons HM, Zebrack BJ, Kirchhoff AC (2016). Social well-being among adolescents and young adults with cancer: a systematic review. Cancer.

[4] O'Sullivan LF, Cheng MM, Harris KM, Brooks-Gunn J (2007). I wanna hold your hand: the progression of social, romantic and sexual events in adolescent relationships. Perspect Sex Reprod Health.

[5] Fincham F, Cui M (eds) (2010) Romantic relationships in emerging adulthood. Cambridge University Press, Cambridge

[6] Janssen SHM, van der Graaf WTA, van der Meer DJ, Manten-Horst E, Husson O (2021) Adolescent and young adult (AYA) cancer survivorship practices: an overview. Cancers (Basel) 13(19). 10.3390/cancers13194847 10.3390/cancers13194847 PMC8508173 34638332

[7] Wettergren L, Kent EE, Mitchell SA, Zebrack B, Lynch CF, Rubenstein MB (2017). Cancer negatively impacts on sexual function in adolescents and young adults: the AYA HOPE study. Psychooncology.

[8] Geue K, Schmidt R, Sender A, Sauter S, Friedrich M (2015). Sexuality and romantic relationships in young adult cancer survivors: satisfaction and supportive care needs. Psychooncology.

[9] Olsson M, Steineck G, Enskär K, Wilderäng U, Jarfelt M (2018). Sexual function in adolescent and young adult cancer survivors-a population-based study. J Cancer Surviv.

[10] Acquati C, Zebrack BJ, Faul AC, Embry L, Aguilar C, Block R (2018). Sexual functioning among young adult cancer patients: a 2-year longitudinal study. Cancer.

[11] World Health Organisation: defining sexual health. https://www.who.int/teams/sexual-and-reproductive-health-and-research/key-areas-of-work/sexual-health/defining-sexual-health. Accessed July 7 2023

[12] Olsson M, Enskär K, Steineck G, Wilderäng U, Jarfelt M (2018). Self-perceived physical attractiveness in relation to scars among adolescent and young adult cancer survivors: a population-based study. J Adolesc Young Adult Oncol.

[13] Saris LMH, Vlooswijk C, Kaal SEJ, Nuver J, Bijlsma RM, van der Hulle T et al (2022) A negative body image among adolescent and young adult (AYA) cancer survivors: results from the population-based SURVAYA study. Cancers (Basel) 14(21) 10.3390/cancers14215243 10.3390/cancers14215243 PMC9655157 36358662

[14] Kirchhoff AC, Fowler B, Warner EL, Pannier ST, Fair D, Spraker-Perlman H (2017). Supporting adolescents and young adults with cancer: oncology provider perceptions of adolescent and young adult unmet needs. J Adolesc Young Adult Oncol.

[15] Frederick NN, Bingen K, Bober SL, Cherven B, Xu X, Quinn GP (2021). Pediatric oncology clinician communication about sexual health with adolescents and young adults: a report from the children’s oncology group. Cancer Med.

[16] Frederick NN, Revette A, Michaud A, Bober SL (2019). A qualitative study of sexual and reproductive health communication with adolescent and young adult oncology patients. Pediatr Blood Cancer.

[17] Perez GK, Salsman JM, Fladeboe K, Kirchhoff AC, Park ER, Rosenberg AR (2020). Taboo topics in adolescent and young adult oncology: strategies for managing challenging but important conversations central to adolescent and young adult cancer survivorship. Am Soc Clin Oncol Educ Book.

[18] Lehmann V, Laan ETM, den Oudsten BL (2022). Sexual health-related care needs among young adult cancer patients and survivors: a systematic literature review. J Cancer Surviv.

[19] Stanton AM, Handy AB, Meston CM (2018). Sexual function in adolescents and young adults diagnosed with cancer: a systematic review. J Cancer Surviv.

[20] Carpentier MY, Fortenberry JD (2010). Romantic and sexual relationships, body image, and fertility in adolescent and young adult testicular cancer survivors: a review of the literature. J Adolesc Health.

[21] Cherven B, Sampson A, Bober SL, Bingen K, Frederick N, Freyer DR (2021). Sexual health among adolescent and young adult cancer survivors: a scoping review from the Children’s Oncology Group Adolescent and Young Adult Oncology Discipline Committee. CA Cancer J Clin.

[22] Graugaard C, Sperling CD, Hølge-Hazelton B, Boisen KA, Petersen GS (2018). Sexual and romantic challenges among young Danes diagnosed with cancer: results from a cross-sectional nationwide questionnaire study. Psychooncology.

[23] Kushal SA, Amin YM, Reza S, Hossain FB, Shawon MSR (2022). Regional and sex differences in the prevalence and correlates of early sexual initiation among adolescents aged 12–15 years in 50 countries. J Adolesc Health.

[24] Tong A, Sainsbury P, Craig J (2007). Consolidated criteria for reporting qualitative research (COREQ): a 32-item checklist for interviews and focus groups. Int J Qual Health Care.

[25] Brinkmann S, Kvale S (2015). InterViews. Learning the craft of qualitative research interviewing.

[26] Oberguggenberger AS, Nagele E, Inwald EC, Tomaszewski K, Lanceley A, Nordin A (2018). Phase 1–3 of the cross-cultural development of an EORTC questionnaire for the assessment of sexual health in cancer patients: the EORTC SHQ-22. Cancer Med.

[27] Frisch M, Moseholm E, Andersson JB, Garugaard C (2019). Sex i Danmark. Nøgletal fra Projekt Sexus 2017–2018 [Sex in Denmark. Key data from Project Sexus 2017–2018].

[28] Malterud K (2012). Systematic text condensation: a strategy for qualitative analysis. Scand J Public Health.

[29] Braun V, Clarke V (2021) Thematic analysis: A practical guide. SAGE, London

[30] Braun V, Clarke V (2014). What can “thematic analysis" offer health and wellbeing researchers?. Int J Qual Stud Health Well-being.

[31] Kvale S, Brinkmann S (2015) Interview: Det kvalitative forskningsinterview som håndværk [Interview: The qualitative research interview as craftsmanship], 3rd edn. Hans Reitzels Forlag, Copenhagen

[32] Hauken MA, Velure GK, Müller B, Sekse RJT (2023) Sexual health and quality of life in cancer survivors with pelvic radiation injuries. Cancer Nurs 10.1097/ncc.0000000000001259 10.1097/NCC.0000000000001259 37449715

[33] Fitz VW, Anderson C, Manuck TA, Mersereau J, Bensen JT, Park J et al (2022) Patient-reported sexual function among young adult cancer survivors. J Adolesc Young Adult Oncol. 10.1089/jayao.2022.0079 10.1089/jayao.2022.0079 36251841

[34] Gu R, Chen H, Wang X, Jin X, Jiang F, Zhao W (2023). The mediating role of appraisal on health-related quality of life in adolescent and young adult cancer survivors. Qual Life Res.

[35] Oveisi N, Cheng V, Brotto LA, Peacock S, McTaggart-Cowan H, Hanley G et al (2023) Sexual health outcomes among adolescent and young adult cancer patients: a systematic review and meta-analysis. JNCI Cancer Spectr 7(6). 10.1093/jncics/pkad087 10.1093/jncics/pkad087 PMC10674049 37878813

[36] Albers LF, Haj Mohammad SF, Husson O, Putter H, Pelger RCM, Elzevier HW (2020). Exploring communication about intimacy and sexuality: what are the preferences of adolescents and young adults with cancer and their health care professionals?. J Adolesc Young Adult Oncol.

[37] Gilbert AL, Rickert VI, Aalsma MC (2014). Clinical conversations about health: the impact of confidentiality in preventive adolescent care. J Adolesc Health.

[38] Bergström C, Lampic C, Roy R, Hedman C, Ahlgren J, Ståhl O (2023). Do young adults with cancer receive information about treatment-related impact on sex life? Results from a population-based study. Cancer Med.

[39] Moules NJ, Estefan A, Laing CM, Schulte F, Guilcher GMT, Field JC (2017). “A tribe apart”: sexuality and cancer in adolescence. J Pediatr Oncol Nurs.

[40] Albers LF, Bergsma FB, Mekelenkamp H, Pelger RCM, Manten-Horst E, Elzevier HW (2022). Discussing sexual health with adolescent and young adults with cancer: a qualitative study among healthcare providers. J Cancer Educ.

[41] Greimel E, Nagele E, Lanceley A, Oberguggenberger AS, Nordin A, Kuljanic K (2021). Psychometric validation of the European Organisation for Research and Treatment of Cancer-Quality of Life Questionnaire Sexual Health (EORTC QLQ-SH22). Eur J Cancer.

[42] Phan J, Laurence V, Marec-Berard P, Cordero C, Riberon C, Flahault C (2023) The place of sick peers in adolescents and young adults with cancer: advantage, disadvantage, and what makes barriers to the encounter. J Adolesc Young Adult Oncol. 10.1089/jayao.2022.0176 10.1089/jayao.2022.0176 36999900

[43] Kaluarachchi T, McDonald F, Patterson P, Newton-John TRO (2020). Being a teenager and cancer patient: what do adolescents and young adults with cancer find valuable and challenging with their friends and cancer peers?. J Psychosoc Oncol.

[44] Kelly D, Pearce S, Mulhall A (2004). ‘Being in the same boat’: ethnographic insights into an adolescent cancer unit. Int J Nurs Stud.

